# Pathophysiology and Clinical Management of Bile Acid Diarrhea

**DOI:** 10.3390/jcm11113102

**Published:** 2022-05-30

**Authors:** Giovanni Marasco, Cesare Cremon, Maria Raffaella Barbaro, Francesca Falangone, Davide Montanari, Federica Capuani, Giada Mastel, Vincenzo Stanghellini, Giovanni Barbara

**Affiliations:** 1 Division of Internal Medicine, IRCCS Azienda Ospedaliero Universitaria di Bologna, 40138 Bologna, Italy; giovanni.marasco4@unibo.it (G.M.); cesare.cremon@aosp.bo.it (C.C.); maria.barbaro2@unibo.it (M.R.B.); davide.montanari8@studio.unibo.it (D.M.); federica.capuani@studio.unibo.it (F.C.); giada.mastel@studio.unibo.it (G.M.); v.stanghellini@unibo.it (V.S.); 2 Department of Medical and Surgical Sciences, University of Bologna, 40126 Bologna, Italy; 3 Medical-Surgical Department of Clinical Sciences and Translational Medicine, University Sapienza, 00185 Rome, Italy; francesca.falangone@uniroma1.it

**Keywords:** bile acid diarrhea, malabsorption, chronic diarrhea, bile acid sequestrants, selenium homotaurocholic acid test

## Abstract

Bile acid malabsorption (BAM) represents a common cause of chronic diarrhea whose prevalence is under-investigated. We reviewed the evidence available regarding the pathophysiology and clinical management of bile acid diarrhea (BAD). BAD results from dysregulation of the enterohepatic recirculation of bile acids. It has been estimated that 25–33% of patients with functional diarrhea and irritable bowel syndrome with diarrhea have BAM. Currently, the selenium homotaurocholic acid test is the gold standard for BAD diagnosis and severity assessment. However, it is an expensive method and not widely available. The validation of the utility in the clinical practice of several other serum markers, such as 7α-hydroxy-4-cholesten-3-one (C4) and the fibroblast growth factor 19 (FGF19) is ongoing. The first-line treatment of patients with BAD is bile acid sequestrants. Patients that are refractory to first-line therapy should undergo further diagnostics to confirm the diagnosis and to treat the underlying cause of BAD. An early and correct diagnosis of BAD would improve patient’s quality of life, avoiding additional diagnostic tests that burden health care systems. Considering the limited availability and tolerability of specific medications for BAD treatment, future research is awaited to identify other therapeutic approaches, such as gut microbiota modulating therapies.

## 1. Introduction

Bile acid malabsorption (BAM) is a cause of chronic diarrhea first recognized by Alan Hofmann in 1967 and called cholegenic diarrhea or cholerheic enteropathy [1]. BAM represents a common but frequently under investigated cause of chronic diarrhea whose prevalence estimate is approximately 1% in the general population [2,3,4]. In addition, it has been reported that approximately 25–33% of unexplained chronic diarrhea is due to BAM [3] and one third of patients treated for irritable bowel syndrome diarrhea-predominant (IBS-D) have idiopathic BAM [5]. BAM results from dysregulation of the enterohepatic recirculation of bile acids (BAs) and its consequent alteration of bile acid production. BAs are synthesized in the liver and are detergent molecules responsible for solubilization of fatty acids and monoglycerides derived by triglyceride lipolysis, thus determining digestion and lipid absorption in the small intestine [1]. The pathophysiology of BAM can be explained by several mechanisms characterized by an excessive level of BAs in the lower gastrointestinal tract, which in turn leads to water and sodium abnormal transport, mucosal damage, mucus secretion, increased lower gastrointestinal motility, and stimulation of defecation [6,7,8]. Gut dysbiosis (i.e., a dysregulation in the intestinal bacterial, fungal and viral ecosystem) has also been recently linked with the gastrointestinal composition and total content of Bas [9,10]. Currently, the gold standard method for identifying BAM and assess its degree of severity is the selenium homotaurocholic acid test (75SeHCAT) using a radio-labelled synthetic conjugated bile acid (23-seleno-25-homo taurocholic acid) which is orally administered, secreted in bile and then reabsorbed in the terminal ileum [11]. The quantification of the severity of BAM is useful to predict the response to therapy, which is currently based on the use of bile acid sequestrants [12]. There is poor recognition by clinicians of this condition resulting in a delayed diagnosis of BAM which is also due to the lack of widely available diagnostic tests [12]. Almost 50% of patients with BAM had experienced symptoms for more than 5 years before being correctly diagnosed [13]. A prompt diagnostic evaluation would contribute to correctly manage the disease and could avoid unnecessary diagnostic test that burden the health care system [14]. The aim of this review is to summarize evidence on the pathophysiology, epidemiology, diagnostic and treatment of BAM to increase the awareness for this condition and its relevance in the management of chronic diarrhea. 

## 2. Search Strategy 

Identification of papers on BAD for this narrative review, was carried out with a literature search up to 10 September 2021 with MEDLINE via PubMed, Ovid Embase, and Scopus using the following medical subject heading (MESH) terms ‘bile acids’ OR ‘bile acids malabsorption’ OR ‘bile acids diarrhea’ OR ‘SeHCAT’ was performed by four authors. Articles more relevant for the topic of this clinical review were selected without language or time restriction; references of selected articles and systematic reviews were also evaluated, when of interest. Pre-clinical, in vivo, and in vitro studies were included only in the pathophysiology section. Disagreements on the relevance of studies selected for inclusion in the review were resolved by a third independent reviewer.

## 3. Pathophysiology

### 3.1. Physiology of Bile Acids Synthesis and Circulation

**Figure 1:**
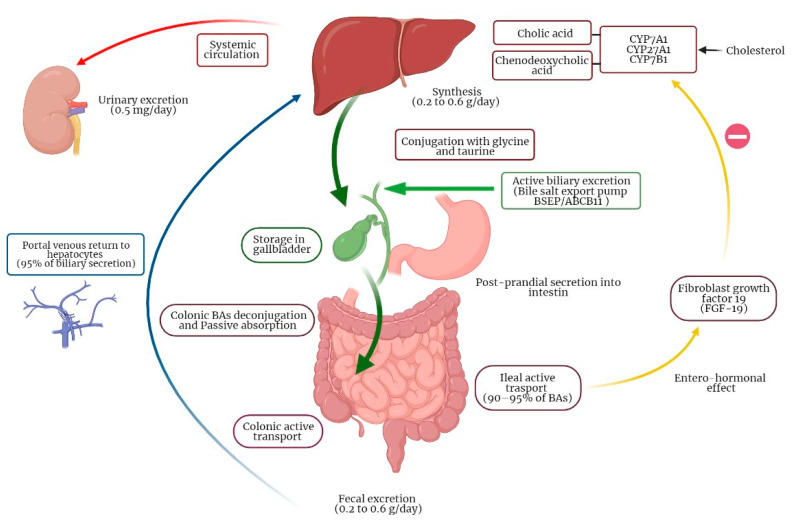
Enterohepatic circulation of bile acids.

BAs are amphipathic molecules synthesized in the liver in a multi-enzymatic process [1]. BAs are obtained from hydroxylation of cholesterol, conjugated with glycine or taurine and finally secreted to bile. This process is the principal metabolic pathway for the catabolism of cholesterol [15]. The primary function of BAs is to facilitate lipids and liposoluble vitamins (A, D, E and K) absorption in the small intestine. BA synthesis in the liver is around 0.5 g per day and a bile acid pool of 3 g is recycled 4–12 times a day. Due to intestinal reabsorption, only 5% of BAs are lost in feces every day (0.2–0.6 g per day) and reintegrated by de novo synthesis by the liver, so that bile acid pool remains constant [16,17]. There are two main pathways for BAs production by the liver: the classic pathway accounting for 90–95% of BAs synthesis and the acidic pathway accounting for up to 10% of BAs production [18,19]. In the classic pathway, the enzyme cytochrome P450 cholesterol-7α-hydroxylase (CYP7A1) represents the limiting enzyme. The acidic pathway is initiated by sterol 27-hydroxylase CYP27A1 catalyzing the transformation of cholesterol into oxysterols, which have cytotoxic properties. Oxysterols are then rapidly hydroxylated by the enzyme oxysterol 7α-hydroxylase (CYP7B1) [20]. BAs produced in the liver are also called primary BAs and include cholic acid (CA), with hydroxyls in position 3α, 7α, 12α, and chenodeoxycholic acid (CDCA), with hydroxyls in position 3α and 7α [21]. After being synthesized, BAs are conjugated with amino acids as glycine and taurine. The final step is the active secretion of BAs from the canalicular membrane of hepatocytes into biliary canaliculi by the transporter bile salt export pump (BSEP/ABCB11) [22]. Bile is stored in the gallbladder during fasting; when food reaches duodenum, cholecystokinin is released and stimulates bile secretion into the small intestine. BAs conjugation increases solubility and impermeability to cell membranes, which allow BAs to form micelles in the small intestine, which in turn interact though their polar side with hydrophobic fatty acids and monoglycerides finally leading to lipid digestion and absorption [10]. In normal conditions, about 90–95% of BAs is reabsorbed in the terminal ileum, while the unabsorbed amount of BAs reaches the large intestine (Figure 1) [23]. BAs reaching the colon interact with gut microbiota, which is responsible for deconjugation, dehydrogenation, 7α-dehydroxylation, and epimerization of primary BAs, producing secondary BAs. In particular, cholic acid (CA) is transformed in deoxycholic acid (DCA), while chenodeoxycholic acid (CDCA) is transformed into lithocholic acid (LCA) and into the tertiary BA, namely ursodeoxycholic acid (UDCA) [16]. BAs colonic reabsorption is a passive process obtained by passive diffusion through enterocytes; at least 50% of the mass of BAs reaching the colon is reabsorbed by diffusion [24]. Intestinal reabsorption, recirculation to the liver and new secretion of BAs by hepatocytes into bile constitutes the enterohepatic circulation of BAs. 

### 3.2. Molecular Mechanisms of BAs Enterohepatic Circulation

BA active reuptake in the terminal ileum requires the apical Na+ dependent bile salt transporter (ABST) and the intestinal BA binding protein (I-BABP), which transports BAs into enterocytes, and the basolateral heterodimeric organic solute transporter (OSTα/β), which in turn secretes BAs into the splanchnic circulation to theF portal vein [16]. In the ileocytes, BAs have an entero-hormonal role. Indeed, high BAs intracellular levels stimulate the production of fibroblast growth factor 19 (FGF19) acting on the nuclear Farnesoid X factor (FXR) [25]. In turn, FGF19 is released in the portal vein and transported to the liver where interacts with the FGF-receptor [26] and with co-receptor klothoβ, resulting in the inhibition of CYP7A1 [27]. An intermediate marker generated by this reaction is 7α-hydroxy-4-cholesten-3-one (C4), which is consequently an indirect marker of BA synthesis [28]. Therefore, BAs synthesis is decreased through a negative feedback mechanism [16]. FGF19 is also involved in the inhibition of gallbladder contraction in the postprandial phase, thus allowing gallbladder filling [29]. 

Besides, BAs can activate receptor G protein-coupled receptor (GPBAR-1, also known as TGR-5) in the intestine the cell surface. TGR5 is an important hormonal regulator in the human body expressed in gallbladder, brown adipose tissue, skeletal muscle, enteroendocrine cells, sinusoidal endothelial cells, bile duct epithelial cells and Kupffer cells, but not in hepatocytes [30,31]. LCA is the strongest natural agonist of GPBAR-1, but this receptor also interact with (un)conjugated deoxycholic acid, such as CDCA, UDCA and cholic acid [32]. In the bile ducts, activation of TGR5 causes gallbladder relaxation and activation of the chloride channel CFTR, improving secretion of bicarbonate resulting in an increase in biliary pH [30]. In this condition a higher proportion of BAs are in the ionized form, which reduces their ability to pass into the biliary epithelium. This process protects bile duct epithelium against the detergent effect of Bas [33]. In Kupffer cells and macrophages, activation of GPBAR-1 has anti-inflammatory properties, through the inhibition of lipopolysaccharide (LPS)-induced cytokine production [32]. Furthermore GPBAR-1 stimulates the secretion of peptide YY (PYY), glucagon-like peptide 1 (GLP-1) and glucagon-like peptide 2 (GLP-2), inducing an anorexigenic effect also influencing glucose metabolism [34].

### 3.3. Pathogenetic Mechanisms of BAM and Clinical Manifestations

**Figure 2:**
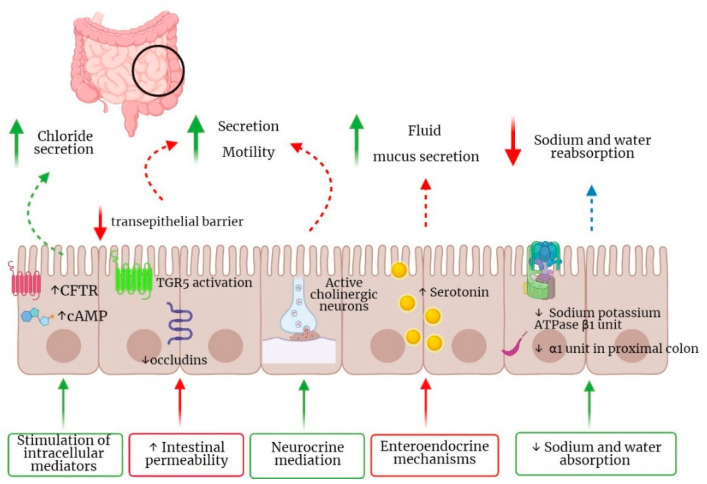
Pathophysiology of diarrhea in bile acid malabsorption. Abbreviations: CFTR: Cystic fibrosis transmembrane conductance regulator; cAMP: cyclic adenosine monophosphate; TGR5: Takeda G protein-coupled receptor 5; ATPase: Adenosine triphosphate-hydrolyzing enzymes.

BAM associated diarrhea (BAD) results from increased colonic motility and water secretion (Figure 2). These alterations are the expression of several molecular mechanisms including the stimulation of intracellular mediators through the increase in cyclic AMP (cAMP), epidermal growth factor receptor (EGFR) and mediators including exchange protein directly activated by cAMP and calcium [35,36]. Moreover, other studies reported an increased expression of aquaporin channels 3 and 8 in rats [37]. In addition, decreased sodium and water absorption in BAD is caused by the reduced expression of sodium potassium ATPase β1 unit in colon and α1 unit in proximal colon [24]. Enteroendocrine mechanisms participating in the pathogenesis of BAD have been recently described, including an increase in serotonin tissue bioavailability with consequent fluid and mucus secretion [38,39,40], and neurocrine mediation through the basal BAs receptor TGR5 which has been shown to be expressed on enteric nerves and enterochromaffin cells, whose activation can regulate small intestinal and colonic motility [28,41]. Moreover, a number of studies suggest an increase in intestinal permeability through detergent and structure-related properties of BAs, TGR5 activation and reduction in occludins [42,43]. Finally, BAM can induce propagating high amplitude colonic contractions [44]. Colonic dysfunction in BAM may also be related to modifications of gut microbiota found in these patients [27]. Indeed, gut microbiota is a major regulator of BAs pool size and composition, which in turn regulate microbiota composition and richness and its characteristics. In particular, gut microbiota is responsible for the transformation of primary conjugated into secondary unconjugated BAs, thus allowing BAs reabsorption through a passive mechanism [9]. Microbiota imbalances have been explored in the context of IBS patients, where significative changes were found. Dysbiosis was reported in IBS patients with different sera and fecal BAs profiles, with an increase in Escherichia coli, Bacteroides and Bifidobacterium in IBS-D patients [45]. Several studies have identified an increased relative abundance of *Firmicutes*, primarily in *Ruminococcaceae* spp. and *Clostridium cluster XIVa* and a reduction in the relative abundance of *Bacteroides* [46,47]. Another study showed that patients with IBS with diarrhea (IBS-D) had an increase in *Escherichia coli* and a decrease in *Clostridium leptum* and *Bifidobacterium*, all influencing BAs pool [48]. In line with these results, other authors showed that 25% of IBS-D patients had an increase in *Clostridia* bacteria, especially *C. scindens* [49]. Indeed, a *Clostridia*-rich microbiota is able to enhance total BAs excretion, which is mirrored by high levels of fecal BAs and serum 7α-hydroxy-4-cholesten-3-one (C4) [49]. Another recent study [50] showed that not only fecal bacterial diversity was reduced in patients with BAD, but that patients with BAD have enriched a bacterial composition enriched in 10 operational taxonomic units (OTUs), such as members of the *Lachnospiraceae family*, *Ruminococcaceae family*, *Bifidobacterium longum*, *Prevotella copri*, *Akkermansia muciniphila* and two members of the *Bacteroides genus* [50]. This imbalance was mirrored by different short chain fatty acids (SCFAs) amounts, with significantly more propionate in BAD [50]. Conversely, another well-designed study concluded that fecal metabolomes, but not microbiomes, can distinguish patients with IBS with vs those without BAM [51]. However, due to the rather low number of experiences in this field, further studies are needed to clarify the clinical meaning of *Clostridia* and the role of metabolomic as potential biomarkers responsible for BAD occurrence and target for therapy [52]. 

Besides, mutations and dysfunctions of constituents involved in the regulation of BAs enterohepatic circulation have been studied as possible mechanisms responsible for idiopathic BAM. In particular, FGF19, produced by the ileal enterocyte, binds Fibroblast growth factor receptor 4 (FGFR4) expressed on the hepatocyte cell membrane. A functional gene *Klothoβ* (KLB) interacts biochemically with FGFR4, allowing FGF19 to trigger intracellular signaling resulting in downregulation of cytochrome P450 7A1 (CYP7A1) activity and suppression of BA synthesis [17]. As matter of fact, defective FGF19 release from the ileum is reported in IBS-D responsive to cholestyramine, consistent with excessive hepatic BA synthesis due to lack of FGF19 signaling causing BA diarrhea [53]. Besides, a well-designed study [54] exploring the etiology of primary BAD, confirmed that its pathogenesis can be mainly due to impairments in ileal FGF19 expression and responsiveness. Indeed, 75SeHCAT retention correlated with the basal ileal transcript expression of FGF19, with the ABST and also with the degree of stimulation by CDCA for FGF19 and I-BABP [54]. Interestingly, no correlations of BAD with genetic variants of FGFR4 and Klotho-β were found [54]. On the other hand, another study [55] identified a DIET1 coding variant which causes an H1721Q amino acid substitution able to increases the levels of FGF19 protein secreted from cultured cells, thus possibly affecting bile acid metabolism. Besides, other authors reported a significant correlation between SNPrs17618244 in the *Klothoβ* gene and accelerated colonic transit in IBS-D [56]. Other authors reported that a gain-of-function variation in GPBAR-1 gene stimulates colonic transit and BA excretion [10]. Finally, genetic mutations of ABST are extremely rare and do not have a clinical impact on BAM, while the reduction in BAs active uptake in the terminal ileum was also previously excluded as a cause of idiopathic BAM.

## 4. Classification

The classification of BAM is based on the causes that lead to malabsorption and diarrhea and includes four categories [2]. Type 1 includes patients with ileal dysfunction and impaired reabsorption of BAs. This can be caused by any disease affecting reabsorption in the terminal ileum, such as Crohn’s disease (CD), ulcerative colitis, ileal resection, radiation ileitis [57]. Type 2 represents idiopathic or primary BAM. It can be considered in some forms of chronic diarrhea in absence of clear gastroenterological diseases or in the diarrhea-predominant IBS [12]. Type 3 is characterized by BAs malabsorption or intestinal dysmotility, due to biliopancreatic diseases, celiac disease, microscopic colitis, small intestinal bacterial overgrowth or cholecystectomy. Finally, type 4 is due to excessive BA synthesis without a clear sign of impaired intestinal reabsorption, e.g., in patients with hypertriglyceridemia or undergoing metformin therapy [58,59]. A recent expert survey from United Kingdom [12] reported that the preferred terminology for classifying BAM is ‘primary or secondary’, followed by ‘types’ and ‘overproduction or malabsorption’. However, since most literature reported BAM classified in ‘types’ we herein use this classification.

### 4.1. Type 1

The prevalence of BAM varies in relation to the type and etiology. Type 1 BAM is secondary to ileal disfunction and it was found in 86% of patients with chronic diarrhea and CD, ileal resection or radiation enteropathy [60]. In CD patients with unresected ileum, BAM prevalence ranges from 11% to 76%, but this prevalence rises to more than 90% in resected CD [61,62]. The correlation between length of resection and bile acid malabsorption is still unclear: while one study reported significantly higher serum C4 levels in CD patients with a resection >70 cm [63], the study by Borghede et al. [61] showed no association. In a study by Gracie et al. [64], BAM prevalence assessed with SeHCAT scanning was 89% in patients who had undergone ileal resection or right hemicolectomy for any reason. Interestingly, BAM prevalence in patients resected for Crohn’s disease was 92%, down to 82% when the resection was performed for other reasons, thus suggesting that CD can be a risk factor for BAM per se. [64] In patients with chronic diarrhea who have undergone ileal resection, a SeHCAT scanning for BAM may not be necessary for the diagnosis of BAM, and an empirical treatment with bile acid sequestrant (BAST) should be administered [61,65]. Chronic diarrhea as consequence of radiotherapy is less common, but a small number of studies have found BAD to be the cause of acute diarrhea during pelvic radiotherapy [66,67]. Despite a very small number of cases, in the study by Borghede et al. [61] three out of three patients with radiation injury showed SeHCAT retention < 15%, and two of them had a retention <10%. Table 1 reports main studies investigating the prevalence of BAM among patients with ileal diseases. 

### 4.2. Type 2

Type 2 BAM is also known as “idiopathic” BAM and manifests clinically as functional diarrhea or IBS-D, since most of these patients have no defect in bile acid absorption. The putative mechanism of this condition is due to an hepatic overproduction of bile acids and an impaired feedback by FGF19 [53]. In a study conducted in 2010 by a group of UK gastroenterologists, only 1% of new cases of patients with chronic diarrhea and 3% of overall chronic diarrhea cases were diagnosed as having BAM [71]. The authors concluded that BAM, particularly type 2, was frequently underdiagnosed and even when considered, most patients did not undergo further diagnostic testing. In a retrospective American study, using 48-h fecal BAs testing, increased BAs excretion was identified in 51% of patients with unexplained diarrhea and, among these, more than 70% had an improvement of their symptom with BAST (compared to 26% of those with normal BA excretion) [72]. In another retrospective analysis of Kurien et al. [70], 38% of patients with chronic diarrhea had evidence of BAM based on SeHCAT result and of these, Type 2 BAM accounted for 28%. A more recent retrospective analysis of Gracie et al. [64] reported that BAM is diagnosed in 50% of patients undergoing SeHCAT and almost 30% of these patients met the criteria for IBS-D. 

In a systematic review by Wedlake et al. [3] almost one third of patients with chronic diarrhea had abnormal fecal bile acid loss using SeHCAT. The authors analyzed 15 prospective studies including patients with IBS-D-like symptoms, finding that the overall BAM prevalence assessed with SeHCAT was 10%. Among these patients, 32% had moderate and 26% mild BAM (SeHCAT retention < 10% and <15%, respectively). In addition, the authors showed a dose–response relationship according to the severity of malabsorption with the treatment with a BAST: 96% of patients with severe BAM responded to cholestyramine, while only 70% of those with mild BAM responded to treatment [3]. Using this data, they estimated that the population prevalence of BAD in the general population was over 1% [3]. Furthermore, in a more recent retrospective study by Borghede et al., 68% of patients with chronic watery diarrhea had abnormal values at SeHCAT scan, of whom 72% had a severe BAM [61]. In other systematic reviews including cohorts of patients either diagnosed with IBS-D [73] or functional diarrhea [74] or with no organic explanation for their chronic diarrhea, about 25% of patients had positive tests for BAD [74]. 

Idiopathic BAM was also found to be associated with post-infective diarrhea. In a study by Niaz et al. [75], 55% of patients with positive SeHCAT test had a history of acute gastroenteritis, while another retrospective study reported that 18% of patients with post-infective diarrhea had BAM responsive to cholestyramine [76]. Interestingly, primary unconjugated BAs (UBAs) levels were high in stool of patients with IBS-D even without overt BAM, while lowest fecal UBAs values (secretory UBAs in particular) were found in IBS with constipation (IBS-C) patients. In support of this, Wong et al. [77] reported that serum C4 levels tend to be higher in patients with IBS-D than IBS-C or healthy volunteers, and this may be related to the fact that patients with IBS-D had greater body mass index, thus synthetizing and excreting higher levels of BAs [77]. These findings support a possible role of BAs in the pathogenesis of IBS, and that the measurement of serum C4 and primary and secondary UBAs in stool, rather than total BAs, may be useful in the diagnostic work-up of IBS [78]. The main studies assessing the prevalence of type 2 BAM are reported in Table 2.

### 4.3. Type 3

Type 3 BAM is related to gastrointestinal disorders not associated with ileal dysfunction, such as post-cholecystectomy diarrhea. Borghede et al. [61] reported that a number of patients who undergone cholecystectomy had diarrhea onset during the same year of cholecystectomy [61]. However, while single studies reported BAM incidence of 68–86% after cholecystectomy [61,64], a systematic review of 25 studies conducted by Farahmandfar et al. [80] showed that only 9.1% of patients who had undergone cholecystectomy developed diarrhea, with two-thirds of them diagnosed with BAM. Among other causes of type 3 BAM, while the mechanisms leading to BAM in post-vagotomy diarrhea are poorly understood [81], BAM associated with chronic pancreatitis seems related to impaired bicarbonate secretion [82]. Besides, BAM in celiac disease seems caused by villous atrophy and impairments in gallbladder and small bowel motor function [83]. However, the reported prevalence of BAM in celiac disease did not differ significantly from that of healthy controls [64,70]. Similarly, BAM in microscopic colitis may be related to villous atrophy, inflammation and collagen deposition in the ileum leading to BAs malabsorption [84]. One study found that 44% of patients presenting chronic diarrhea with collagenous colitis had a SeHCAT retention < 10%, of whom 78% showed a rapid response to treatment with BAST [85]. Consequently, other authors suggests that treatment with BAST should be considered in all patients with microscopic colitis, even in the absence of a positive SeHCAT test [86]. Some authors previously reported a category separate from type III, named Type 4 BAM, resulting from an increased synthesis of BAs without a clear source of impaired bile acid reabsorption, as reported during treatment with metformin or in patients with hypertriglyceridemia. While there are no relevant studies in literature investigating type 4 BAM epidemiology, an old questionnaire-based study reported that 20% of patients treated with metformin had accelerated bowel transit times [87] A recent study also suggests that non-alcoholic fatty liver disease (NAFLD) is associated with increased hepatic BAs production and diarrhea, resulting in elevated serum C4 in this patients [88]. Finally, obesity, together with hypertriglyceridemia and a decrease in HDL cholesterol were shown to be associated with an increase in bile acid synthesis and idiopathic forms of BAD [89,90]. Taken together these cases often identified as type 4 BAM, indicate that this is a metabolic disorder rather than a type of BAM separate from type 3. The main studies reporting the prevalence of type 3 BAM are reported in Table 3. 

## 5. Clinical Manifestations 

Patients with BAM complain an increased frequency of watery chronic diarrhea (80%) and fecal urgency (85%), associated with abdominal discomfort, which can include abdominal pain, bloating and fecal incontinence [13]. According to a recent study, patients with IBS-D with BAD reported a greater impact on bowel and somatic symptoms and quality of life compared with those without BAD, mainly due to more severe and frequent diarrhea along with urgency, which was supported by an increased need of toilet proximity [91]. In addition, patients with BAD reported an increased use of antidiarrheals, bile acid binders and antacid secretory agents than those without [91]. Affected individuals may also report systemic symptoms including fatigue, dizziness and feeling of fainting [65]. After treatment with BASTs, at least 50% of patients refer improvement or resolution of both gastrointestinal and systemic symptoms [13]. It should be underlined that the chronicity of symptoms and their impact on daily habits and social life, often accompanied by a missed or late diagnosis, can compromise the quality of life and with a consequent development of depressive symptoms [2,13].

## 6. Diagnosis

### 6.1. Selenium HomotauroCholic Acid Test (75SeHCAT)

75SeHCAT is the test with the highest diagnostic accuracy for BAM [92]. SeHCAT have high sensitivity and specificity (94 and 100%, respectively) in discriminating different subsets of patients with chronic diarrhea compared to healthy subjects [93]. In comparison to other diagnostic tests, 75SeHCAT showing an average reported sensitivity and specificity of 87.32 and 93.2%, followed by serum 7α-hydroxy-4-cholesten-3-one with 85.2 and 71.1%, respectively [94]. 

Homotaurocholic acid, a synthetic conjugated bile acid, is labeled with the 75 selenium (Se) isotope mimicking the action of an endogenous BA. This radiotracer is absorbed through the intestine, excreted by the liver and reabsorbed in the terminal ileum (enterohepatic circulation) [95]. The patient should discontinue the treatment with BAST for a few days and fast for 4 h before the ingestion of the 75SeHCAT capsule [95]. The first baseline acquisition with gamma camera is taken within 3 h from ingestion, whereas the second one after 7 days. Low retention of radiolabeled BAs indicates fecal loss. [95] Based on the percentage of retained radioactive selenium, BAM can be distinguished as severe (<5%), moderate (5–10%) and mild (10–15%). The severity of BAM has been shown to influence the response to therapy with BAST [96]. Moreover, being a 7 day-test, it is less affected by day-to-day variation and dietary effects [4]. However, the different thresholds for assessing BAM presence in published studies may have affected its reported accuracy [3]. To overcome these limits, Wedlake et al. [3] summarized available results in a systematic review including 43 studies for a total of 1223 IBS patients, finding that 75SeHCAT retention thresholds were as following: 10% of patients with <5%, 27% with <10% and 13% with <15% of retention. In a more recent systematic review [92], the 75SeHCAT retention rate cut-off <10% was most widely accepted for increasing the diagnostic accuracy of BAM. Furthermore a 75SeHCAT retention < 10% correlates with a faster colonic transit time [67]. Other limits are that 75SeHCAT is not available in many countries apart from few tertiary referral centers and it requires a nuclear medicine department, specialized equipment and trained personnel. Moreover, considering that the test consists of two phases of scintigraphic recording (at day 0 and after 7 days), it is time consuming for patient. Finally and not negligible, it foresees radiation exposure [92].

### 6.2. Hour Fecal Bile Acid Test

This test is the first option to diagnose BAD in countries where 75SeHCAT is not available [78]. It requires measurement of total and individual BAs in the feces of patients with chronic diarrhea using high performance liquid chromatography (HPLC). Patients must consume a high fat diet (100 g/day) and collect stool for the next 48 h, which makes the test quite cumbersome and not particularly attractive, particularly for patients. Diagnostic criteria of BAD are the follows: total fecal BAs ≥ 2337 µmol/48 h or primary BAs (CDCA and CA) > 10%, or total fecal BAs ≥ 1000 µmol/48 h associated with primary BAs > 4% [97]. Primary fecal BAs (CDCA and CA) are significantly higher in patients with BAD and correlate with stool frequency and consistency [78,89]. Indeed, it has been reported that patients with BAD have higher stool weight compared to those with chronic diarrhea without BAD or healthy volunteers [97]. Regarding the accuracy of this test, elevated primary BAs alone or in combination with total fecal BAs >1000 µmol/48 h have a similar diagnostic accuracy as total fecal BAs alone >2337 µmol/48 h in stool of patients with BAD [2,97]. In a recent retrospective study including 986 patients with chronic diarrhea who underwent 48-h fecal BA measurement, Vijayvargiya et al. reported that only 26% of patients had elevated total fecal BAs, whereas 46% of patients had fecal primary BAs > 10%; these data suggested that the measurement of total fecal BAs alone may miss a subgroup of patients with BAD [97]. However, differently from 75SeHCAT, [3] currently there are no randomized clinical trials that evaluate the response to therapy with BAS in relation to the different composition of BAs in the stool (total *vs* primary fecal BAs) [2].

### 6.3. Fasting Serum 7α-hydroxy-4-cholesten-3-one (C4) and Fibroblast Growth Factor 19 (FGF19)

C4 is a serum biomarker that represents a direct measure of hepatic BA synthesis [2]. It is a metabolic intermediate involved in the synthesis of BAs from hepatic cholesterol [98]. C4 is measured by HPLC in the serum of patients with suspected BAM [92]. C4 concentration >48.3 ng/mL are considered diagnostic of BAM [92].

FGF19 is a hormone released by ileal enterocytes after stimulation of nuclear farnesoid X receptors (FXR), usually induced by BAs reabsorption [99], and it provides negative feedback for BA synthesis in hepatocytes [78]. Therefore, it represents an indirect measure of ileal bile acid reabsorption [78,89]. FGF19 is measured by enzyme-linked immunosorbent assay (ELISA) in the serum of patients with chronic diarrhea [100]. In order to avoid potential changes induced by meal consumption, FGF19 has to be assessed during fasting [98]. The cut-off value for FGF19 with the highest diagnostic accuracy for BAM diagnosis has been reported to be ≤145 pg/mL [92]. There is a close correlation between C4 and FGF19 in the pathophysiological mechanism of BAM. Indeed, when BAs are absorbed in the ileum, the nuclear FXR receptors induce FGF19 transcription and synthesis. Then, FGF19 binds hepatic receptors causing the decrease in C4 and the inhibition of the conversion of cholesterol to BAs. Therefore, when BAs are reabsorbed, a greater amount of FGF19 is released from the enterocyte, resulting in a decreased serum level of C4, finally reflecting the decrease in hepatic BAs synthesis [101]. In conclusion, decreased FGF19 and elevated C4 are diagnostic for BAD [101]. Both C4 and FGF19 have diurnal variations, with a gradual increase after 9 am [102]. For this reason, their samples should be performed before 9 am, to avoid false results [2]. In comparison to the accuracy of other diagnostic tests, fasting serumC4 > 48.4 ng/mL has a sensitivity of 90% and specificity of 79% [103], and fasting serum FGF19 < 145 pg/mL a sensitivity of 58% and specificity of 84%, compared to 75SeHCAT < 10% [104]. Instead, compared to total fecal BAs > 2337 µmol/48 h, fasting C4 > 52.5 ng/mL (rated on 184 healthy volunteers) has a sensitivity of 25% and a specificity of 90%, and fasting FGF19 < 61.7 pg/mL a sensitivity of 32% and a specificity of 78% [98]. In clinical practice, the performance of the C4 assay could be used as screening test to rule out BAM, due to its high negative predictive value (NPV). In fact, compared to the 75SeHCAT test, C4 test has NPV of 98%, and positive predictive value (PPV) of 74% [105]. Data regarding FGF19 reported that, compared to values of 75SeHCAT < 10%, the NPV and PPV of FGF19 as a marker of BAM ranged from 50 to 80% (mean 63.8%). A recent prospective study conducted on 152 patients, reported that the NPV and PPV of FGF19 ≤ 145 pg/mL were 82 and 61% [104]. Besides, using a FGF19 cut-off <60 ng/L in a group of 466 patients with chronic diarrhea related to Crohn’s ileitis, FGF19 had a sensitivity and specificity of 80 and 68% [63]. An important limitation of C4 evaluations is that some pathological conditions, such as liver disease (cholestatic disease with hypertriglyceridemia, AST or ALT > 2× upper limit of normal) or therapy with medications able to modify BA synthesis (i.e., statins), could determine false-positive or false-negative results [106]. Moreover, it is unclear if age, emotional conditions, or environmental factors such as shift work or jet-lag may play a role in the circadian rhythm, thus influencing the synthesis of BAs [105]. Finally, also C4 test requires specialized equipment and personnel [105]. Regarding the use of FGF19 in clinical practice, it is an easy, non-invasive and relatively not expensive diagnostic technique, which can be assessed by commercially available ELISA kits [107]. However, its levels show significant variations due to meal consumption and is necessary measure its values during fasting [107].

### 6.4. BAST Empirical Trial

BAST empirical trial is not recommended to diagnose BAM, despite its use is justified in the absence of other diagnostic tests [108]. However, there is little evidence regarding the comparison of SeHCAT testing vs empiric trial of BAST and most guidelines reached this conclusion as a conditional recommendation taking into account several factors [108]. It consists in testing the response to cholestyramine or other bile acid sequestrants (i.e., colestipol and colesevelam) in patients with chronic diarrhea, without a previous diagnosis of BAM. These patients could be not motivated to continue the treatment with unpalatable BAST and may discontinue it, resulting in a false-negative cholestyramine trial [109]. Conversely, a positive diagnostic test has the advantage to improve patient’s compliance to treatment, encouraging modification of dosage of cholestyramine when ineffective, or switch to colesevelam to overcome cholestyramine intolerance [109].

## 7. Bile Acids Diarrhea Treatment 

### 7.1. Dietary Modifications

Low-fat diet with less than 20% of daily energy intake provided from fat, associated with BASTs, is effective in improving gastrointestinal symptoms of BAD such as abdominal pain, nocturnal defecation, urgency, flatulence, stool consistency and frequency [110,111]. A prospective evaluation conducted on 42 patients with gastrointestinal symptoms due to BAM, showed that patients, after dietary intervention (mean dietary fat intake reduced to 42 g/day) reported a significant reduction for urgency, bloating, lack of control, bowel frequency (*p* ≤ 0.01) [111]. More recent prospective data in 114 patients with 7-day scan retention <20%, demonstrated that, after dietary intervention, there was a statistically significant improvement in abdominal pain and nocturnal defecation (*p* = 0.001), while there was no amelioration of bowel frequency, urgency, flatulence, belching, borborygmi and stool consistency [110]. Therefore, according to the abovementioned studies, a dietary intervention is an effective approach in patients with symptomatic BAM and should be routinely considered.

### 7.2. BAs Sequestrants

Cholestyramine, colestipol and colesevelam are BASTs available either in powder or tablet formulations. These molecules are positively charged non-digestible resins which bind BAs in the intestinal lumen forming an insoluble complex that is eliminated in stools [27]. BASTs are effective in improving abdominal symptoms, stool frequency and consistency [112]. Patients with severe BAD diagnosed by 75SeHCAT showed a better response to BASTs than patients with higher BAs retention [12]. Consequently, quantifying the severity of BAM is useful to predict BAST response [12]. 

Cholestyramine is the most widely used BAST [10]. A randomized clinical trial [113] compared cholestyramine *vs* hydroxypropyl cellulose in patients with chronic watery diarrhea and SeHCAT 7-day retention ≤ 20% [113]. The cholestyramine group (4 g twice daily for 8 weeks) reported higher decrease in watery stools number (*p* = 0.048). In another randomized trial assessing cholestyramine efficacy in BAD, patients with 75SeHCAT retention of 10% or 20% reported response rates of 40% and 53.8%, respectively [27]. However, cholestyramine is poorly tolerated because of unpalatability and for common gastrointestinal side effects such as constipation, abdominal pain, bloating, fullness, nausea and flatulence [114]. Side effects finally results in a low compliance to therapy. Moreover cholestyramine sometimes causes transient hypertriglyceridemia [115] and could interfere with the intestinal absorption of drugs such as tetracycline, penicillin G, cyclosporine, statins (pravastatin and fluvastatin), levothyroxine, olmesartan, furosemide, hydrochlorothiazide, propranolol, phenobarbital, warfarin, digoxin, glyburide, glimepiride glipizide and oral contraceptives. Thus, it is reasonable to administer other medications 1 h before or 4 h after cholestyramine intake [116]. Moreover, high doses of cholestyramine (greater than 32 g/day) may be associated with malabsorption of fat-soluble vitamins and hemorrhagic diathesis and osteomalacia have also been reported with cholestyramine, due to an impairment of vitamin K and vitamin D absorption [117]. Rarely, particularly in patients with renal insufficiency and patients on aldosterone antagonists such as spironolactone [118], cholestyramine may cause hyperchloremic metabolic acidosis [119,120] due to its ability to exchange chloride anions for BAs in the lumen of the small intestine, resulting in fecal excretion of BAs. 

Colesevelam, is a water-insoluble polymer with a binding affinity to BAs 4–6 times stronger than cholestyramine. Moreover, it is related to higher patient compliance due to lower incidence of side effects (constipation, dyspepsia and nausea) and fewer clinical interactions [121]. In a single center study conducted on IBS-D patients, colesevelam was associated with significantly increased total fecal BA excretion, thus improving stool consistency and reducing the number of bowel movements per week [100]. Similarly, in a randomized, double-blind, placebo-controlled trial including 24 patients with IBS-D undergoing colesevelam at the dosage of 1.875 g, twice daily, an increase in stool consistency scores (Bristol Stool Form Scale) [122] of 0.56 ± 0.06 units per unit change in colonic transit at 24 h was also reported [100]. In another open-label study conducted on patients with high 48-h stool BAs excretion, colesevelam 1875 mg administered twice daily for 10 days, led to an amelioration of stool consistency and an increased stool excretion of sequestered BAs [112]. In addition, other authors reported also a reduction in median number of liquid stools from 5 to 2 per week [123]. Colesevelam showed efficacy even in IBS-D patients, where a reduced emptying of the ascending colon was reported [100]. Finally, it should be underlined that after BAST treatment, the total fecal BAs and serum C4 were higher than baseline. Indeed, BAST can inhibit the physiological negative feedback of hepatic BA synthesis, resulting in increased BA production [60].

### 7.3. Farnesoid X Receptors (FXR) Agonists

The activation of the FXR pathway by agonist drugs such as the obeticholic acid (OCA) (which is chemically 6-ethyl CDCA) is able to induce the transcription of FGF19 and the inhibition of CYP7A1,which is the first and rate-limiting enzyme in bile acid synthesis [124]. FXR agonists attenuate chloride secretion to calcium and cAMP-dependent agonists in the intestinal epithelium, with improvement of diarrhea demonstrated both in vitro and in vivo [125]. Consequently, FXR agonists lead to increased serum FGF19, and decreased serum C4 and fecal BAs [27]. These molecules have been previously proposed to treat cholesterol gallstone disease and cholestasis [25]. Recently, FXR agonists have been used in patients with BAM. In particular an open-label, single-center pilot study evaluated mechanisms, safety and symptom response of OCA in three groups of patients with BAD studied over 6 weeks [124]. The authors reported that, in relation to the type of BAM, two weeks of therapy with OCA hesitated in a statistically significant increase in FGF19 in primary and secondary BAD, due to reduced BAs synthesis [124]. Another recent double-blind, multicenter, randomized study evaluated tropifexor, which is a non-bile acid FXR agonist affecting BA metabolism and colonic transit in patients with primary BAD, finding that tropifexor (60 µg once daily) had acceptable safety and tolerability in this patients [126].

### 7.4. Microbiota Modulation

Considering the ability of BAs to modulate bowel functions and their tight correlation with gut microbiota, an additional interest has been paid to the evaluation of gut microbiota in BAM in order to employ microbial modulating therapies in these patients [78,89]. Fecal BA pool in IBS-D patients is different from that of healthy subjects [97] also due to dysbiosis, especially to the reduction in genera *Ruminococcaceae* [127] and the increase in *Clostridia* bacteria (e.g., *C. scindens*) which correlated with the levels of fecal BAs and serum C4 in IBS-D patients [49]. However, to date no definitive data are available regarding the beneficial effect of gut microbiota modulating therapies also used in IBS such as diet, pre- and probiotics and eubiotics as rifaximin, in the context of BAM.

## 8. Conclusions and Future Strategies

BAM is an under investigated cause of chronic diarrhea. In patients with persistent symptoms of watery diarrhea, ileal disease or following gastrointestinal surgery, bile acid diarrhea should be suspected and investigated. Difficulties in early diagnosis due to limited availability of diagnostic test, in particular 75SeHCAT, have been widely reported. Considering the limited availability of specific medications for the treatment of this condition and the poor tolerability and compliance of patients for these drugs, future research is awaited to identify other therapeutic approaches, such as gut microbiota modulating therapies.

## Figures and Tables

**Table 1:** Studies investigating the prevalence of bile acid malabsorption type I.

Study	Year	Nation	Total Number of Patients with Ileal Disease, n	Patients with BAM, n (%)	Clinical Feature	Diagnostic Method	Treatment
Nyhlin et al. [68]	1994	UK	51	34 (67%)	Diarrhea	SeHCAT retention < 10%	Cholestyramine
Smith et al. [69]	2000	UK	81	60 (74%)	Diarrhea	SeHCAT retention < 10%	Antidiarrheals BAS
Borghede et al. [61]	2011	Denmark	87	77 (88%)	Diarrhea	SeHCAT retention < 15%	Cholestyramine
Kurien et al. [70]	2011	UK	47	40 (85%)	Diarrhea	SeHCAT retention < 10%	n/a
Lenicek et al. [63]	2011	Czech Republic	232	112 (48%)	Inflammatory bowel disease-related	Serum C4 FGF19	n/a
Gracie et al. [64]	2012	UK	90	62 (69%)	Diarrhea	SeHCAT retention < 15%	n/a

Abbreviations: number, n; bile acid malabsorption, BAM; United Kingdom, UK; selenium homotaurocholic acid test, SeHCAT; bile acid sequestrants, BAS; not available, n/a; fibroblast growth factor 19, FGF19.

**Table 2:** Studies investigating the prevalence of bile acid malabsorption type II.

Study	Year	Nation	Total Number of Patients, n	Patients with BAM, n (%)	Clinical Feature	Diagnostic Method	Treatment
Ford et al. [79]	1992	UK	74	20 (27%)	Diarrhea	SeHCAT retention < 15%	Cholestyramine
Smith et al. [69]	2000	UK	197	65 (33%)	IBS-D	SeHCAT retention < 10%	Antidiarrheals BAS
Borghede et al. [61]	2011	Denmark	114	68 (60%)	Diarrhea	SeHCAT retention < 15%	Cholestyramine
Kurien et al. [70]	2011	UK	102	36 (34%)	Diarrhea	SeHCAT retention < 10%	n/a
Gracie et al. [64]	2012	UK	77	21 (27%)	IBS-D	SeHCAT retention < 15%	n/a
Vijayvargiya et al. [72]	2020	USA	936	476 (51%)	Diarrhea	48-h fecal BA excretion	Cholestyramine Colesevelam Colestipol

Abbreviations: number, n; bile acid malabsorption, BAM; United Kingdom, UK; selenium homotaurocholic acid test, SeHCAT; bile acid sequestrants, BAS; irritable bowel syndrome with diarrhea, IBS-D; not available, n/a; United States of America, USA; fibroblast growth factor 19, FGF19.

**Table 3:** Studies investigating the prevalence of bile acid malabsorption type III.

Study	Year	Nation	Total Number of Patients, n	Patients with BAM, n (%)	Clinical Feature	Diagnostic Method	Treatment
Ford et al. [79]	1992	UK	30	24 (80%)	Cholecystectomy	SeHCAT retention < 15%	Cholestyramine
			11	4 (36%)	Vagotomy		
Ung et al. [85]	2000	Sweden	27	12 (44%)	Collagenous colitis	SeHCAT retention < 10%	Cholestyramine Colestipol
Borghede et al. [61]	2011	Denmark	36	31 (86%)	Cholecystectomy	SeHCAT retention < 15%	Cholestyramine
			12	4 (33%)	Microscopic colitis		
Kurien et al. [70]	2011	UK	31	21 (68%)	Cholecystectomy	SeHCAT retention < 10%	n/a
			12	4 (33%)	Celiac disease		
			1	1 (100%)	Vagotomy		
			11	3 (27%)	Connective tissue disease		
			8	2 (25%)	Pancreatic insufficiency		
Gracie et al. [64]	2012	UK	76	52 (68%)	Cholecystectomy	SeHCAT retention < 15%	n/a
			6	1 (17%)	Celiac disease		
			18	6 (33%)	Collagenous colitis		
			6	3 (50%)	Lymphocytic colitis		
Farahmandfar et al. [80]	2012	UK	55	36 (65%)	Post-cholecystectomy diarrhea	SeHCAT	Cholestyramine
Appleby et al. [88]	2019	UK	92	11 (12%)	NAFLD	Serum C4 FGF19	n/a

Abbreviations: number, n; bile acid malabsorption, BAM; United Kingdom, UK; selenium homotaurocholic acid test, SeHCAT; not available, n/a; United States of America, USA; non-alcoholic fatty liver disease, NAFLD; fibroblast growth factor 19, FGF19.

## Data Availability

The data presented in this study are openly available in Medline and Embase.
